# Dramatic Reduction in Diarrhoeal Diseases through Implementation of Cost-Effective Household Drinking Water Treatment Systems in Makwane Village, Limpopo Province, South Africa

**DOI:** 10.3390/ijerph15030410

**Published:** 2018-02-27

**Authors:** Resoketswe Charlotte Moropeng, Phumudzo Budeli, Lizzy Mpenyana-Monyatsi, Maggy Ndombo Benteke Momba

**Affiliations:** Department of Environmental, Water and Earth Sciences, Arcadia Campus, Tshwane University of Technology, P/B X 680, Pretoria 0001, South Africa; Bphumu@gmail.com (P.B.); monyatsil@tut.ac.za (L.M.-M.)

**Keywords:** diarrhoeal disease, HWTS implementation, water and sanitation

## Abstract

The main purpose of this study was to implement cost-effective household water treatment systems in every household of Makwane Village for the reduction of diarrhoeal diseases. These household water treatment systems were constructed with locally available materials and consisted of the biosand zeolite-silver impregnated granular clay filters and the silver-impregnated porous pot filters. During the study period (April 2015 to September 2015), the entire village had 88 households with a population size of 480. Prior to the implementation, a survey was conducted and results revealed that 75% (360/480) of the Makwane residents suffered from diarrhoeal disease and the majority of the cases were reported in children that were less than five years of age. Out of the 480 participants, 372 (77.5%) from 70 households accepted the installation of the systems (intervention group) and 108 (25.5%) from 18 households were reluctant to use the systems (the control group). To date, in the intervention group, only 3.8% (14/372) of participants reported cases of diarrhoea. In the control group, 57.4% (62/108) participants reported cases of diarrhoea and most of the episodes of diarrhoea were reported in children of less than five years old (85%), followed by the group aged ≥56 years (75%). The findings of the current study unequivocally demonstrated that the BSZ-SICG and SIPP filters were able to reduce the incidence of diarrhoea by 96.2%. These findings further demonstrate the importance of household water treatment systems (HWTS) interventions in rural areas to bring about meaningful reductions in diarrhoeal diseases by providing safe potable water.

## 1. Introduction

Target 7.C of Goal 7 of the Millennium Development Goals (MDGs) for water is to reduce by half the proportion of people without sustainable access to safe drinking water by 2015 [1]. While the MDG target of 88% coverage for access to improved drinking water was met in 2010, the report by WHO/UNICEF Joint Monitoring Programme (JMP) [2], highlighted that 748 million people still depend on unsafe drinking water sources and approximately half of them live in sub-Saharan Africa.

Lack of basic services such as safe drinking has a great impact on individuals, households, communities, and the country as a whole. It has been reported that nearly 2 million people suffer devastating waterborne diseases on an annual basis, and these even result in high mortality for certain cases [3], among which an estimated 6000 children under the age of five years are the first victims [4] and the majority of deaths occurring in developing countries. Approximately 88% of these diseases have been reported to be attributable to unsafe drinking water supply, inadequate sanitation, and poor hygiene [5]. It has been estimated that waterborne diseases are the second most common cause of death in children under the age of five years and the majority of deaths occur in sub-Saharan Africa and Southern Asia [6,7,8].

Access to safe drinking water supply is currently one of the most complex challenges facing a number of rural communities of South Africa, especially for those living in scattered rural areas. A report issued by the Statistics South Africa in 2013 [9] pointed out that access to safe drinking water for poor households has increased to 71.6% in 2011 [10]. This report also revealed a decrease in the incidence of diarrhoea per 1000 children less than five years of age, down from 121.4 per 1000 children in 2004 to 102.1 per thousand children in 2011. In spite of this decrease, a recent report shows that diarrhoea still remains one of the leading causes of morbidity and mortality in under-five children in South Africa; however, the true burden of childhood diarrhoea is not accurately known [11]. 

Due to an increase in the number of deaths that are caused by diarrhoea reported every year as a result of contaminated drinking water, point-of-use (POU) water treatment systems are encouraged in rural areas. A home-made water treatment system can produce safe drinking water using any easily reached water sources, such as rivers, streams, ponds, and canals, regardless of their quality and allows for people to adapt to any seasonal disparity. Implementation of household water treatment systems (HWTS) in rural areas without access to safe drinking water sources allows the treatment of drinking water at POU and also improves the quality of the water. This household drinking-water supply intervention can be considered as a quick and sustainable solution to address the burden of disease caused by lack of access to safe drinking water in scattered rural communities of South Africa.

As part of a Water Research Commission project undertaken between 2010 and 2012 by the Tshwane University of Technology Water Research Group (TUT WRG) a range of homemade water purification devices were trialled under laboratory conditions and were then found to be cost-effective [12,13]. The five types of low-cost filters that seemed to hold the greatest potential for South African conditions included: the silver-impregnated porous pot (SIPP) filter (a TUT Product), the ceramic candle filter (CCF), the biosand filter (BSF), a modified biosand filter with zeolite (BSF-Z—a TUT product), and a bucket filter (BF). With the exception of the CCF, these low-cost filters were designed and built by the TUT WRG. Among these HWTS devices, two filters were found to have higher performance in terms of pollutant removals. The SIPP filter was found to be efficient in achieving complete removal of waterborne pathogens from a variety of water sources; however, it could not deliver sufficient volume of water as its flow rates ranged from 0.05 to 2.49 L/h. In contrast, the BSF-Z filter, which had a higher flow rate up to 19.2 L/h showed waterborne pathogen removal rates ranging between 1 and 4.8 log (90–99.99%) and required a disinfection step to render the filtered water safe for drinking [12,14]. These two HWTS devices therefore required some modifications prior to their implementation in rural communities. The BSF-Z and SIPP filters could play a vital role in providing safe drinking water to rural communities without access to improved drinking water sources

The aim of the present study was threefold: firstly, to enhance the performance of the BSF-Z and SIPP filters in terms of pathogen removal and flow rate; secondly, to deploy these devices in every household of the Makwane Village and investigate their performance while they are in use in homes; and, thirdly, to ascertain their performance in eradicating or reducing the burden of diarrhoeal diseases. 

## 2. Methodology

### 2.1. Modification of BSF-Z and SIPP Filters

**Figure 1:**
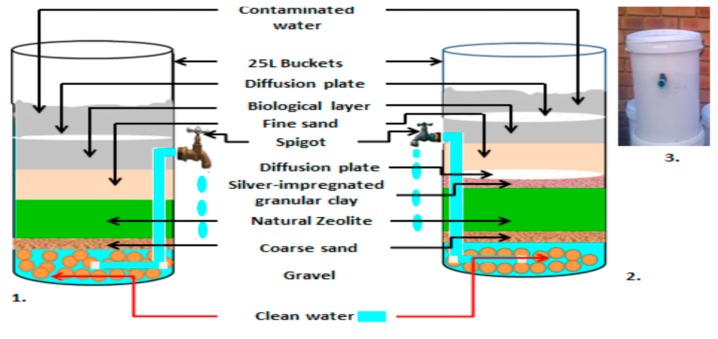
Schematic representation of biosand filters: 1. BSF-Z; and 2. modified BSF with zeolite and silver-impregnated granular clay; 3. BSZ-SICG filter (7 mm gravel; 0.95 mm coarse sand; 3 mm natural zeolite; silver-impregnated granular clay; 0.15 mm fine sand).

**Figure 2:**
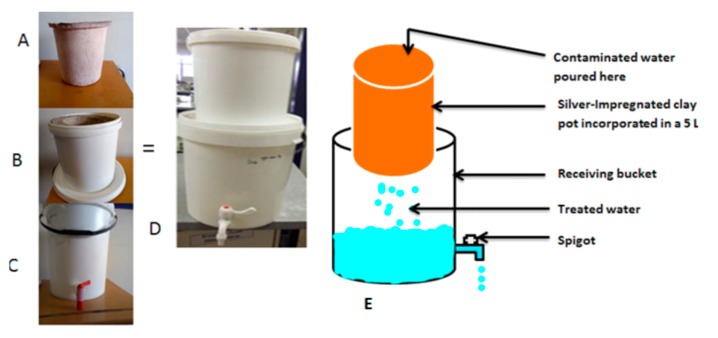
(**A**) Silver-impregnated porous pot (SIPP) filter; and (**B**) 5 L bucket with silver impregnated clay pot inside; (**C**) 10 L receiving bucket; (**D**) Complete SIPP filter; and, (**E**) Schematic representation of SIPP filter.

The project team worked closely with Cermalab cc (Materials Testing Laboratory, CSIR, Pretoria, South Africa) for manufacturing of the biosand zeolite-silver impregnated granular clay (BSZ-SICG) and silver-impregnated porous pot (SIPP) filters to enhance their performance. The SIPP filter was modified in terms of the flow rate, which increased from 2.49 L/h to 27.5 L/h. The BSZ-SICGs were modified from the biosand filters with zeolite (BSF-Z) that were previously constructed by [12]. A layer of silver-impregnated granular clay, which was prepared by mixing ball clay, sawdust, paper fibre, and silver nitrate (AgNO_3_), and moulded into small granulates prior to firing was added to the BSF-Z filter to form a BSZ-SICG filter. The size of these filters was scaled down to 25 L in order to ensure that the filter would not take up too much space in homes of rural communities. Moreover, the spigot was elevated to allow for the filters to maintain a 5 cm of biological layer above the surface of fine sand to prevent it from drying out. The flow rate of this filter was increased from 19.2 L/h to 38.6 L/h. With these improved flow rates, both HWTS devices achieved the required volume of 25 L/person/d. Fifty-five (55) SIPP filters and thirty-five (35) BSZ-SICG filters were thereafter manufactured between December 2014 and March 2015. All of these HWTS devices were tested in the laboratory in terms of pathogen removal and the leaching of silver into the treated drinking water. They were found to produce safe drinking water that complied with SANS 241-1:2011 [15] and also with the WHO [16] guideline values of 0.1 mg/L silver in final drinking water that is meant for human consumption. Figure 1 provides a schematic representation of the modifications made to the BSF-Z filter to produce the BSZ-SICG filter and Figure 2 shows a schematic representation of a SIPP filter. 

### 2.2. Deployment of the Filters in Makwane Village

#### 2.2.1. Description of the Study Area

**Figure 3:**
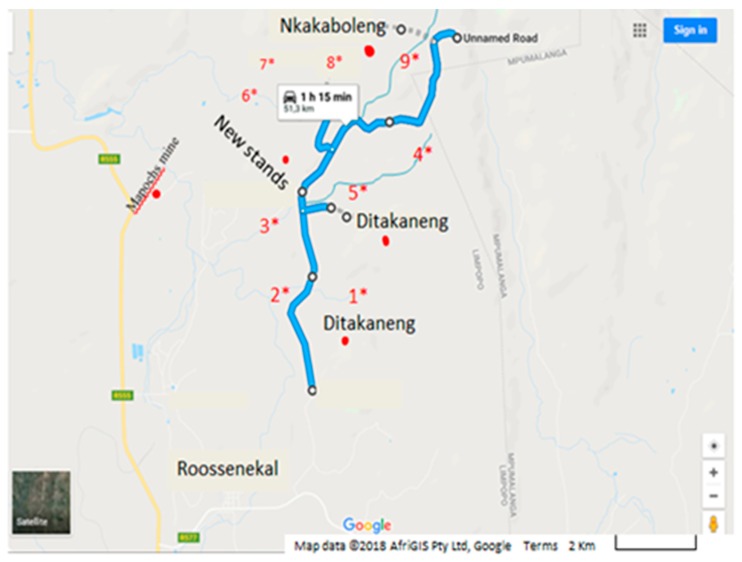
Map of Makwane Village showing all the sections and the surrounding streams/rivers where some of the samples were collected in addition to water collected from households. Source: [17].

Makwane Village was the target rural community without access to improved drinking water sources; the village is situated in the Elias Motsoaledi Local Municipality of the Limpopo Province. This Village comprises four sections: Nkakaboleng, New Stands, Lepururu and Ditakaneng which are surrounded by streams (Figure 3). The village had 88 households and a population of 480 with one primary school during the study period. Residents of this village have limited infrastructure and live in close proximity to domestic animals (goats, cows, sheep, dogs, etc.), which drink from and defecate in the same primary water sources that are used by the community for drinking and domestic purposes.

#### 2.2.2. Ethical Approval

The study was conducted in accordance with the Declaration of Helsinki, and approved by the Faculty of Science Research Ethics Committee (FCRE) at the Tshwane University of Technology (TUT), where the study was registered (Ref: FCRE 2015/03/040 (2) (SCI)). Access to Makwane Village was obtained through the local pastor and community leaders. Furthermore, authorisation to conduct the study was also obtained from the municipal manager, the municipal councillor, and the local municipal committee. All of the households that were selected for participation were given informed consent forms to sign (Supplementary File 1) at the beginning of the project. The project expectations and respective obligations by both the participants and investigators were explained and any questions were answered. The participants were not subjected to risks of any kind as a result of the project. The investigators provided feedback and information to the participants at regular intervals, conducting the project in the most open manner possible.

#### 2.2.3. Deployment of the Filters in Households of Makwane Village

**Figure 4:**
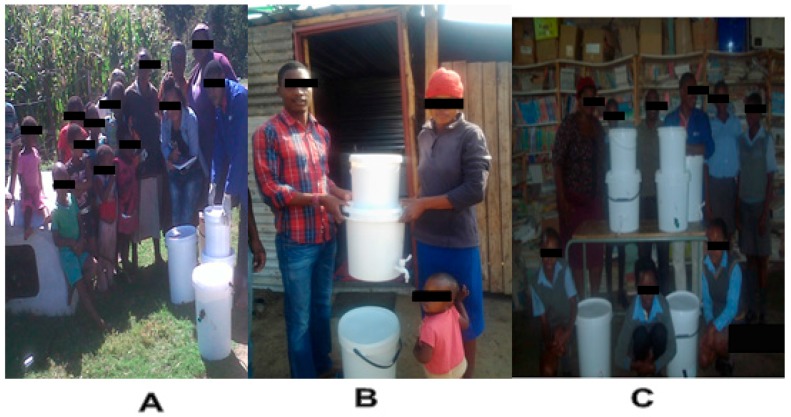
Deployment of the household water treatment systems (HWTS) devices in Makwane Village: (**A**,**B**) deployment in some of the households of Makwane community; (**C**) Deployment at a local primary school in Makwane Village.

A total of 90 HWTS devices (35 BSZ-SICG and 55 SIPP) were transported to Makwane Village subsequent to modification for implementation. Two systems were deployed at a local school in Makwane village, while 88 systems were implemented in every household of the Makwane community. Out of 88 households, 18 households were later used as controls in the study because these householders were reluctant to use the systems. All of the households equipped with HWTS devices were also provided with a 25 L improved storage container with a tap installed 5 cm from the base of the bucket. The intervention phase of the study included weekly household observations from April 2015 to September 2015. Householders were advised to use only treated water from HWTS for cooking and drinking purposes. All of the households that participated in the intervention and the control groups continued to provide detailed information on a weekly basis of diarrhoeal disease incidents. In each household, water quality parameters, such as turbidity and bacterial (pathogenic *E. coli*) counts were measured every week during the first three months and thereafter every second week of the last three months. Figure 4 below illustrates how the research team worked hand-in-hand with the Makwane community during deployment of the HWTS devices.

### 2.3. Water Quality Assessment

Samples of drinking water were collected during household visits from both control households and households with HWTS (intervention households). Control households provided a sample of untreated water used for drinking from their storage containers, while intervention households provided drinking water samples from the storage containers (untreated water), directly from BSZ-SICG and SIPP filter outlet tubes and treated water that had been stored in improved storage containers for drinking. Turbidity was tested using a Hach 2100P Portable turbidity Meter (Eutech Instrument Turbidimeter TN-100, Thermo Scientific, Johannesburg, South Africa) during sampling.

#### 2.3.1. Cuture-Based Methods for the Isolation of Presemptive Pathogenic *E. coli*

In order to determine the presence of presumptive pathogenic *E. coli*, water samples were collected in a 250 mL sterile bottle and transported on ice to the in-field laboratory and analyses were performed within two hours using culture-based techniques. Briefly, MacConkey agar with sorbitol (Merck, Johannesburg, South Africa) was used for the detection of *E. coli* O157H:7 (enterohaemorrhagic *E. coli*—EHEC) and MacConkey agar without sorbitol (Merck, Johannesburg, South Africa) was used for the detection of other pathogenic *E. coli* (enteropathogenic *E. coli*—EPEC; enterotoxigenic *E. coli*–ETEC; enteroaggregative *E. coli*—EAEC and enteroinvasive *E. coli*—EIEC). Individual colonies were randomly selected based on their sizes, shape, and colour, and inoculated in 2 mL Brain Heart Infusion Broth (BHIB). The samples were then incubated overnight at 37 °C upon which they were archived by 20% glycerol and transported on ice packs to the TUT laboratory for further analysis. 

The archived isolates were streaked onto nutrient agar plate (Merck, Johannesburg, South Africa) and incubated at 37 °C for 24 h. The colonies were further purified at least three times employing the same methods and medium before Gram-staining. Subsequently, oxidase tests were conducted on those colonies that were Gram-negative. Thereafter, the oxidase-negative colonies were inoculated into a 2 mL Eppendorf tube containing nutrient broth and incubated at 37 °C for 24 h. The preservation of these colonies was done with 20% glycerol and kept at 20 °C until they were used for molecular studies. 

#### 2.3.2. Molecular Identification of Pathogenic *E. coli*

A total of 250 oxidase-negative isolates were used for the molecular study. Each individual isolate was streaked onto nutrient agar and incubated at 37 °C for 24 h. The total genomic DNA from the bacterial isolates was subsequently extracted by boiling method described by Theron et al. (2000) with some modifications. Briefly, a loopful of colonies was transferred into 1.5 mL of nuclease-free water containing 7 µL of Triton X-100. The samples were then vortexed and boiled at 99 °C for 30 min. The DNA was collected through centrifugation at 12,000 rpm for 15 min. The genomic DNA was quantified using the NanoDrop^TM^ 2000 spectrophotometer (Thermo Scientific, Johannesburg, South Africa).

The DNA templates were subjected to multiplex PCR with specific primers (Table 1), as previously described by [18], for the detection of the following virulence genes of *E. coli*: *stx1* (Shiga-toxin 1 of EHEC), *bfpA* (structural gene for the bundle-forming pilus of EPEC), *estA* (ST) (heat-stable toxin of ETEC), *aaiC* (chromosomal secreted protein of EAEC), and *ipaH* (invasion plasmid antigen H of EIEC). The PCR assay was performed with a 25 µL reaction mixture containing 2.5 µL of template DNA, 12.5 µL of DreamTaq Green PCR Master Mix (2X), which is a ready-to-use solution containing DreamTaq DNA polymerase (2X DreamTaq Green Buffer, dATP, dCTP, dGTP, and dTTP, 0.4 mM each, and 4 mM MgCl_2_) and 0.2 µL of each primer. Nuclease-free water was added to a final volume of 25 µL. The amplification cycles consisted of an initial DNA denaturation at 94 °C for 7 min, followed by 39 cycles of denaturation at 94 °C for 30 s, primer annealing at 57 °C for 30 s, extension at 72 °C for 1 min, and a final extension at 72 °C for 10 min. Negative controls, substituting DNA template with nuclease-free water (Inqaba, Pretoria, South Africa), were included in all PCR runs. The DNA extracted from *E. coli* ATCC 25922 (Quantum Biotechnologies, Johannesburg, South Africa) was used as a positive control. The PCR products (8 µL) were evaluated with a 1.5% (wt/vol) agarose gel (Life Technologies, Johannesburg, South Africa) at 120 mV for 60 min. A molecular marker (100 bp DNA ladder; Inqaba, Pretoria, South Africa) was run concurrently. All of the results were captured using a gel documentation system (Syngene, Cambridge, UK). 

### 2.4. Surveillance of Episodes of Diarrhoea before and after Implementation

One elderly person in each household was identified as the primary respondent during the recruitment period. A structured 10-min interview was conducted weekly in Sepedi (a local language of the Makwane community) in collecting information on the respondents’ personal and domestic hygiene practices, sanitation systems, and the episodes of diarrhoea for all the members of the household during the previous seven days. Diarrhoea was defined as three or more loose or watery stools containing blood or mucus during a period of 24 h and also the frequency of visits to the toilet by a person with diarrhoea within a 24-h period. The diarrhoea reduction percentage (% DR) after HWTS implementation was calculated as follows:

(1) $$\%\;\mathrm{DR}\;=\;\frac{\left(number\;of\;households\;with\;or\;without\;HWTS-diarrhoeal\;cases\right)}{\left(number\;of\;households\;with\;or\;without\;HWTS\;\right)}\;\times \;100$$

### 2.5. Turbidity Removal Efficiency

The level of turbidity in water samples before and after filtration was determined using a portable turbidity meter (2100P Hach, Process Instrument (Pi), Burnley, UK). Turbidity reduction percentage achieved by all of the HWTS devices was calculated according to [19], as follows

(2) $$\%\;\mathrm{turbidity}\;\mathrm{reduction}=\frac{\left(\mathrm{turbidity}\;\mathrm{unfiltered}-\mathrm{turbidity}\;\mathrm{filtered}\right)}{\left(turbidity\;unfiltered\right)}\;\times \;100$$

### 2.6. Monitoring of Silver Leached from BSZ-SICG and SIPP Filters

The concentration of silver in water treated by BSZ-SICG SIPP filters was monitored on a monthly basis throughout the study period. Samples were sent to the Department of Chemistry (Tshwane University of Technology, Pretoria, South Africa) for analysis of leached silver. The SPECTRO ARCOS ICP spectrometer (SPECTRO ANALYTICAL INSTRUMENTS (PTY) LDT, Kempton Park, Johannesburg, South Africa) was used to detect and determine the concentration of silver in treated water samples.

### 2.7. Efficiency of the HWTS Devices in Removing Pathogenic E. coli Strains from Makwane Water Sources

The efficiency of the HWTS devices in removing pathogenic *E. coli* was determined by comparing the concentrations of all the target pathogenic strains before and after treatment. Enumeration of presumptive *E. coli* before and after treatment was done by standard methods. The log reductions were calculated using the equation below and were converted to the percentage *E. coli* removed [19]:

(3) $$\%\;E.coli\;\mathrm{removal}=100-\;\frac{\left(\mathrm{survival}\;\mathrm{counts}\right)}{\left(initial\;counts\;\right)}\;\times \;100$$

### 2.8. Statistical Analysis of Data

Paired and independent *t*-tests were run on Excel using the XLSTAT statistical software (XLSTAT 2017: Data Analysis and Statistical Solution for Microsoft Excel) to determine any significant differences between control and intervention groups. Pearson’s correlation coefficient (*r*) was used to determine the correlation between *E. coli* removal efficiency and leaching silver from SIPP and BSZ-SICG filters. The effect of using the BSZ-SICG and SIPP filters on diarrhoeal disease was determined by comparing the prevalence of diarrhoeal disease for all the households in each group. In this study, the classification of diarrhoeal diseases was carried out according to the WHO (2006) definition of three or more loose or watery stools in any 24-h period. Unpaired *t*-tests were used to compare geometric mean *E. coli* concentrations and turbidity between groups.

## 3. Results

### 3.1. Demographic Information of the Study Area

**Figure 5:**
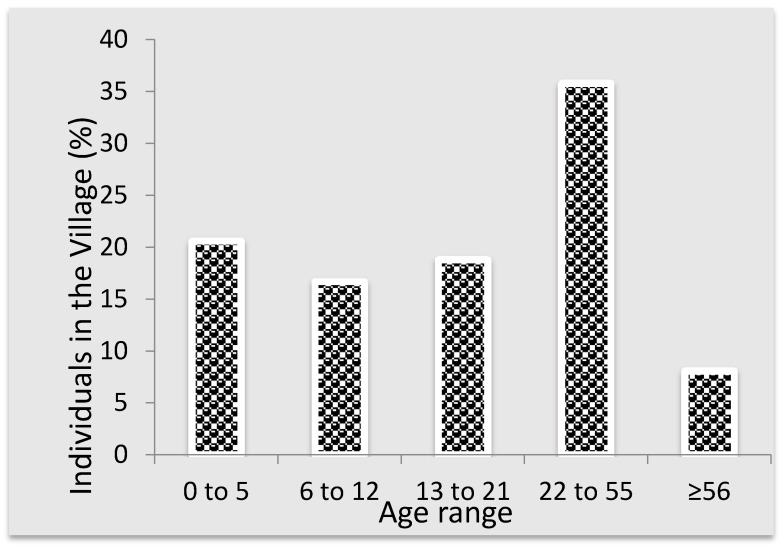
Population distribution of Makwane Village by major age groups.

During the study period (April 2015 to September 2015), the entire village had 88 households with a population size of 480. Among this population, the most dominant groups were between 22 and 55 years (35.8%) and children less than five years old (20.6%). The demographic information of the Makwane Village community during the study period is provided in Figure 5.

### 3.2. Characteristics of Study Population Based on Episodes of Diarrhoea, and Water and Sanitation Facility Per Household

Prior to HWTS implementation in Makwane Village, a higher incidence of diarrhoeal diseases was reported (Table 2). The statistical analysis showed a significant difference between households that never experienced diarrhoea (25%) and those that had experienced diarrhoea (75%) with a *p*-value of 0.000176115. Moreover, all of the households in Makwane Village use water directly from the available sources without prior treatment. The majority of households (70.5%) use water obtained directly from the streams (surface water), while 13.6% use water obtained from springs/wells. About 9.1% of the households have boreholes in their yards and they use water from the boreholes without any treatment. In addition, during rainy seasons 6.8% of the households use roof-harvested rain water. Out of 88 households, 52 (59.1%) households have access to proper sanitation facilities, while 36 (40.9%) had no access to sanitation facilities and the difference was not statistically significant (*p* = 0.369174254). Table 2 shows the characteristics of the study population based on episodes of diarrhoea prior to HWTS implementation, as well as water and sanitation per household. 

### 3.3. Water Quality Analysis

#### 3.3.1. Average Mean *E. coli* Reduction, Turbidity Reduction, Temperature and pH of Untreated Water from Control Households and Treated Water from Intervention Households in Makwane Village

The average Log10 *E. coli* and percentage removal efficiency together with turbidity reduction, temperature, and pH, are summarised in Table 3 below. Much higher average *E. coli* counts (4.3830 Log10 CFU/100 mL) were observed in the control households, than those observed in the intervention households (0.4770 Log10 CFU/100 mL) that were using BSZ-SICG filters. The difference was statistically significant with a *p*-value of 0.000004201. Although *E. coli* counts were detected in the drinking water of the intervention households that were using the BSZ-SICG filters, none of the pathogenic strains were detected. The water that is produced by the SIPP filter (% *E. coli* reduction = 100%) and the BSZ-SICG filter (% *E. coli* reduction = 89.1%) was consistently free of the target bacteria regardless of the quality of the water source. The average turbidity of the untreated water in the control households was found to be 168 nephelometric units (NTU), which exceeded the WHO guideline limits. All of the households equipped with HWTS devices showed a significant reduction in turbidity when compared to the control households (*p* = 0.000025949). The SIPP filters produced drinking water that had the lowest turbidity (0.85 NTU) and was within the recommended limits (SANS 241: <1 NTU; EPA: ≤1; WHO: 5 NTU), while the BSZ-SICG filters produced water with a turbidity of 2.34 NTU, which was within the WHO guideline limits. Untreated water was found to have the highest temperature (25.1 °C), while the treated water had lower temperatures, namely 19.8 °C and 22.5 °C for BSZ-SICG and SIPP filters, respectively. All of the temperature values were within the limits set by both SANS 241 and WHO. The pH values of both untreated and treated water were also within the EPA guideline limits (6.5–8.5). Untreated water had a mean pH value of 8.2, whereas the treated water had a mean pH value of 7.8 and 7.5 for BSZ-SICG and SIPP filters, respectively.

#### 3.3.2. The Leaching of Silver Ions into Water Treated by SIPP and BSZ-SICG Filters over the Study Period (April 2015–September 2015) versus *E. coli* Removal Efficiency

**Figure 6:**
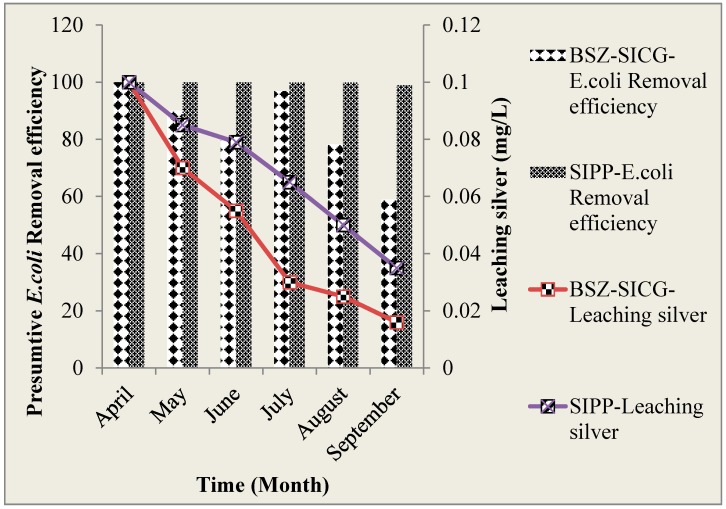
The concentration of silver leached from SIPP and BSZ-SICG filters into treated water versus *E. coli* removal efficiency.

After manufacturing and prior to deployment of the HWTS in Makwane village, the leaching silver was above the WHO recommended limit. In an attempt to reduce the concentration of the silver in HWTS, the SIPP filters were soaked in containers containing municipal treated tap water, while for the BSZ-SIGC, this water passed through the filters until the recommended silver concentration limit was reached. The HWTS devices were subsequently deployed in Makwane village and evaluated for silver leaching in the treated water for 12 weeks between April and September 2015. Figure 6 shows the concentration of silver leached from SIPP and BSZ-SICG filters into treated water throughout the study period. Slower depletion of the silver concentration was observed in the SIPP filters than in the BSZ-SIGC filters with the concentration of 0.100 mg/L to 0.045 mg/L and 0.100 mg/L to 0.016 mg/L for SIPP and BSZ-SIGC filters, respectively. Moreover, the silver leached from both SIPP and BSZ-SIGC filters into the treated water was within the guideline limits set by the EPA (2012), which is 0.10 mg/L. It was observed that the presumptive *E. coli* removal efficiency decreased with a decrease in the concentration of silver that leached into the treated water.

#### 3.3.3. Pearson’s Correlation between Presumtive *E. coli* Removal Efficiency and Silver Leached into Water Treated by BSZ-SIGC and SIPP Filters at POU

The results revealed a strong positive correlation between *E. coli* and silver leached into the water treated by the BSZ-SICG filter (*r* = 0.678627405) and the SIPP filter (*r* = 0.705154424). However, the relationship between silver leached and *E. coli* concentration in the water treated by BSZ-SIGC and SIPP filters was not statistically significant (*p* = 4.59594 × 10^−13^ and *p* = 7.48692 × 10^−6^, respectively).

#### 3.3.4. Amplification of Pathogenic *E. coli* Strains of by Multiplex PCR

**Figure 7:**
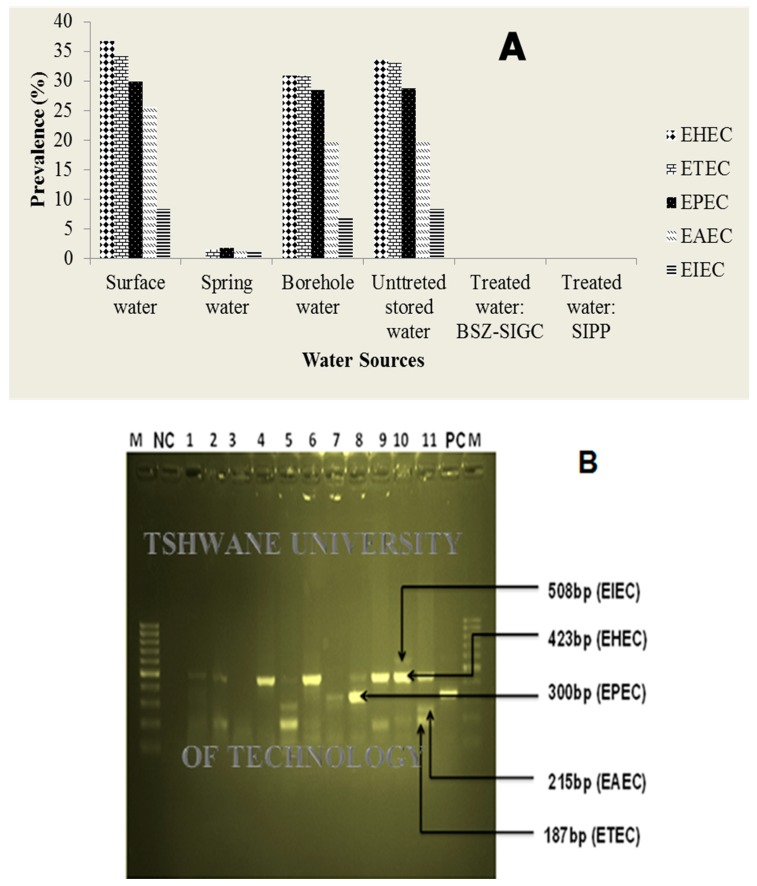
(**A**) Prevalence of pathogenic stains of *E. coli* from different water sources over the study period; and (**B**) agarose gel electrophoresis of PCR products showing several genes representing *E. coli* pathotypes using published primer (M—molecular marker (100 bp); NC—negative control; PC—positive control; and, 1 to 11—samples from different sources).

All of the water samples that were presumptively positive for five targeted pathogenic *E. coli* strains were randomly selected for molecular characterisation. However, none of the five pathogenic strains of *E. coli* were detected in water samples after using the BSZ-SICG and SIPP filters (Figure 7A). The *stx1* gene coding for EHEC was the most frequently detected gene in surface water, borehole water, and storage containers with a prevalence of 36.7%, 30.9%, and 33.6%, respectively. The *ipaH* gene coding for EIEC was the least isolated with a prevalence of 8.3% (surface water), 1.2% (spring water), 6.7% (borehole water), and 8.3% (storage containers). All of the results are presented in Figure 7A below. A typical result after agarose gel electrophoresis of PCR products showing several genes representing *E. coli* pathotypes isolated from water sources of Makwane Village is given in Figure 7B.

### 3.4. Diarrhoeal Disease Incidence per Age Group and Stool Consistency Subsequent to HWTS Implementation in Makwane Village

Subsequent to HWTS implementation in Makwane Village, episodes of diarrhoea were found to be more prevalent in control households than in intervention households. The majority of the diarrhoea episodes were reported in children that were less than five years old from both the control and intervention households with a prevalence of 85% and 5.1%, respectively. The difference was statistically significant with a *p*-value of 0.000000105. The second highest incidence of diarrhoeal disease was observed in the elderly group of ≥56 years with a prevalence of 75% (control households) and 11.11% (intervention households). The statistical analysis showed a significant difference with a *p*-value of 0.0005018. Conclusively, 57.4% of the respondents in control households reported episodes of diarrhoea, while only 3.8% of the respondents in the intervention households reported episodes of diarrhoea. The incidence of diarrhoea was thus reduced by 96.2% in the intervention households. In general, watery diarrhoea was the main type of diarrhoea reported by Makwane Village residents. In control households, 62 cases of diarrhoea were reported (57.4%), most of which were watery diarrhoea (69.3%), followed by mucus diarrhoea (21%) and bloody diarrhoea (9.7%), while 14 cases of diarrhoea were reported in intervention households (3.8%), all of which (14/14) were watery diarrhoea. All of the results are summarised in Table 4.

## 4. Discussion 

According to [2], most of the rural communities in sub-Saharan Africa still face significant challenges in gaining access to improved drinking water and are struggling to meet the MDG targets for water and sanitation. It has been estimated that about 16 million people in South Africa have no access to adequate water and sanitation [20], and the lack of such services significantly contribute to diarrhoeal diseases worldwide. To address these issues, a study on the effect of HWTS implementation was undertaken in one of the rural villages in South Africa; these HWTS devices were deployed in every household of the village that was willing to participate and their performance was assessed in terms of their ability to reduce the burden of diarrhoeal diseases in the community. Prior to HWTS implementation in Makwane Village, 75% of the householders had reported episodes of diarrhoea and it was also found that the majority of the households (70.5%) in Makwane use water obtained directly from the river/surface without prior treatment (Table 2). The episodes of diarrhoea that were reported in the study area could be due to a lack of improved water sources and proper sanitation (Table 2), as it has been previously reported that a lack of such facilities contributes significantly to diarrhoeal diseases with higher cases reported in children aged five years and less [5,6,7,8,11]. Therefore, the high number of children under the age of five years old in this study indicates the potential vulnerability of this community. Successful HWTS implementation will therefore be a solution to reduce the burden of diarrhoeal diseases in the study area, as improved household water management and storage ensures that the drinking water is microbiologically safe at the point of use.

### Water Quality

Surface water is an important supply of drinking water for many populations worldwide, principally in rural areas. Therefore, it is imperative that it must be judiciously managed and protected. Safe drinking water must have an acceptable quality that complies with physical, chemical, and bacteriological parameters that are set by [21,22,23]. The surface water used by the Makwane community was shown to have mean pH and temperature values that are within the limits set by EPA (2012) for drinking and domestic purposes, which is 7.0–8.12 for pH and ≤25 °C for temperature (Table 3). The cause of a decrease in temperature can be attributed to the second law of thermodynamic, which state “in a closed system, the potential energy of the system will always be less than that of the initial state” [24]. The HWTS implemented in Makwane were closed systems and there is a possibility that they can exchange heat with the surroundings, so when untreated water is filtered it will exchange temperature with the content of the systems as a result the temperature of the final product will be less than the initial temperature. Even though the pH and the temperature of the Makwane water sources cannot cause any health risk to the community, their surface water sources were found to have high average turbidity levels of up to 268 NTU, which by far exceeded the limit (5 NTU) that is set by the [23] guidelines. Turbidity in drinking water is caused by particulate matter that may be present in the water source such as clay, silt, organic matter, inorganic matter, plankton, and other microscopic organisms [25]. Moreover, high levels of turbidity in the water are associated with poor water quality and promote the survival of microorganisms [25]. The turbidity reduction in intervention households (households using BSZ-SICG and SIPP systems) was statistically significant as compared to untreated water in the control households (Table 3), suggesting that HWTS implementation in Makwane Village households had improved the turbidity of the water. The average percentage reduction in turbidity obtained during the period of the study was 98.6% (2.34 NTU) and 99.5% (0.84 NTU) for BSZ-SICG and SIPP filters, respectively. The average turbidity level achieved complies with the turbidity limits set by [21,23], and they also compare well to previous findings in which the percentage reduction in turbidity ranged from 88% to 99% for ceramic candle filters [26]. The results of this study in terms of percentage reduction in turbidity levels are also similar to the results reported by [27], with average turbidity reduction levels of between 83% and 99% being achieved for ceramic silver-coated water filters. Another similar study conducted in South Africa by [12,14] reported that a range of locally produced point-of-use water filters, including BSF and SIPP, consistently reduced the turbidity of surface water in the laboratory with an average turbidity of up to 98%. 

In order to determine whether the leached out silver from the BSZ-SICG and SIPP filters complies with EPA 2012, guidelines (0.1 mg/L), the silver concentration was measured in the filtered water prior to and after HWTS implementation over the study period (six months). The mean silver concentration leached in filtered water from both HWTS devices was found to be within the limits recommended by [6,22]. Results for both silver leached and the *E. coli* removal efficiency are clearly shown in Figure 6. The findings of this study revealed that the silver level in the BSZ-SICG filter was depleted much faster compared to SIPP filters. However, the difference is not clearly understood as in both cases silver is incorporated within the clay. 

This study also assessed the efficiency of the BSZ-SICG and SIPP filters in removing pathogenic *E. coli* strains from various water sources of Makwane Village. A significant improvement in household water quality was documented in this study for pathogenic *E. coli* strains (EHEC, ETEC, EPEC, EIEC, and EAEC) during the six-month assessment period (Figure 7). Since the proportion of the population in this village without access to improved drinking water is high (Table 2) and the water that was assessed was found to have high levels of microbial contamination (4.3830 Log10 CFU/100 mL of *E. coli*) and often very turbid (Table 3), this study further provides important evidence regarding HWTS implementation in sites where access to safe water is inadequate. 

During the study period, the BSZ-SICG filter demonstrated an average reduction of 89.1% for *E. coli* (Table 4) in Makwane water sources. Moreover, none of the pathogenic strains of *E. coli* was detected in water treated by the BSZ-SICG filter (Figure 7). These results were found to be in line with the results of other studies in some countries, which reported 60% to 100% *E. coli* removal efficiencies of the BSF filters both in the field and laboratory [28,29]. A laboratory-scale study conducted by [14] showed a reduction in indicator bacteria of between 60% and 100% and 90% and 100%, respectively, in the biosand filter with zeolite (BSZ-SICG) and the SIPP filter. The findings of this study also compare well to those of previous studies reported by different researchers that showed that the BSF could achieve 90% to 99.99% *E. coli* removal [30,31]. A higher reduction of the target bacteria by the BSZ-SICG filter may be due to the presence of silver-impregnated clay granules in the system, which is reported to have bactericidal properties [32,33]. However, it was also observed that even though the silver concentration in the BSZ-SICG filter system was low, the systems continued to remove pathogenic *E. coli* from water. This could be attributed to the unknown pore size of the BSZ-SICG systems. In addition, it was reported that the removal of bacteria by biosand filters at the initial stage occurs by sedimentation and straining, and that with frequent use of these filters, the removal efficiencies increased, sometimes up to 99.99% [31]. However, this was not found to be the case in this study, as the pathogen removal efficiency of the BSZ-SICG filter was found to decrease with frequent use (down to 78.9%). This might have been due to the fine sand that managed to penetrate the diffusion plate with some bacteria attached and moved to the bottom layer of the BSZ-SICG filter system. Silver ions did not have any effect on the bacteria attached to the fine sand as it was also shown to deteriorate with frequent use of the systems; hence, at low concentration, Ag^+^ does not have an antibacterial effect. As a result, the quality of treated water deteriorated. However, an increase in the efficiency to remove pathogenic *E. coli* of up to 96.9% was observed in the BSZ-SICG filters after they had been washed during the third month of being used. Therefore, the maintenance of these systems is important and, depending on the volume of water filtered, they need to be washed every third month of use to ensure good quality drinking water.

The SIPP filter also demonstrated a total reduction of pathogenic *E. coli* (Table 4 and Figure 7). These findings are similar to the results of a study done by [34], where the author attempted to determine the highest possible reduction of target pathogenic bacteria by a silver-impregnated clay-pot filter which resulted in 99.99% reduction. Other similar studies conducted both in the laboratory and in the field for BSF, SIPP, and other filtration technologies showed the pathogen reduction ranging between 90% and 100% [12,14,31,35,36,37,38]. Greater removal efficiency (100%) of target pathogenic bacteria by the SIPP filter in this study might have been achieved by silver (Ag) nanoparticles, which were embedded within the clay during manufacturing [39]. A study by [40] also revealed that the Ag-impregnated pot was significantly more effective in removing *E. coli*, when compared to the control pot without silver.

In this study, an overall and significant reduction (96.2%) in the incidence of diarrhoeal disease was documented in households that were using BSZ-SICG and SIPP filters as compared to the control households. These results suggest greater or comparable reduction in diarrhoeal disease in comparison to randomised control trials of ceramic water filters in SA and Zimbabwe, which demonstrated an 80% reduction in diarrhoeal diseases [36]. These results also compare well to studies of other HWTS devices such as the concrete BSF in Cambodia, the Dominican Republic, and Kenya which demonstrated a 47% reduction in diarrhoeal disease (both Cambodia and Dominican Republic), and a 54% reduction in Kenya, in children under the age of five years [35,41,42]. A related study conducted in Ghana also showed a 60% reduction in diarrhoeal diseases in households that were requested to use the plastic BSF as compared to control households [43]. In general, the results for diarrhoeal disease reduction in intervention households as compared to control households (Table 4) observed in this study suggest greater reductions compared to those obtained during trials of concrete BSF and ceramic filters in other regions of the world. 

In addition, as children under the age of five years were a subgroup of interest in this study, data on diarrhoeal disease were also analysed by age groups (Table 4). The results showed a major reduction (90.5%) in diarrhoeal disease in children less than five years old in the BSZ-SICG and SIPP intervention as compared to the control households and the difference was statistically significant (*p* < 0.01). These results suggest a greater reduction in diarrhoeal disease when compared to the 44% reduction in diarrhoeal disease in children under the age of five years reported in Cambodia [41]. Therefore, in rural areas were people still mainly rely on untreated water for drinking and domestic purposes, the implementation of BSZ-SICG and SIPP filters is substantiated by the results of this study.

## 5. Conclusions

This study investigated the performance of two cost-effective household water treatment systems in reducing the burden of diarrhoeal diseases, while they were in use in homes of the Makwane village. Prior to their deployment, they were subjected to some modifications to enhance their performance. Both the BSZ-SICG and SIPP filters were found to be effective in removing pathogenic *E. coli* from water sources used by the community of the Makwane Village. Furthermore, they have demonstrated their capability to reduce the incidence of diarrhoeal diseases by 96% in the Makwane Village community of the Limpopo Province, South Africa. These cost-effective technologies can be recommended in rural areas without access to improved drinking water supply. In addition, research on HWTS technologies shall attempt to measure health impacts in more objective ways that can assist in eliminating bias. These may include incorporating diagnostic procedures to detect intestinal infections in children in order to compare the organisms found in water with those found in stool samples.

## Figures and Tables

**Table 1:** Oligonucleotides used in this study for the amplification of several genes representing *E. coli* pathotypes.

Primer Name	Sequences 5’→3’	Target Genes	Size	Reference
EHEC-423	F-TGGAAAAACTCAGTGCCTCT- R-CCAGTCCGTAAATTCATTCT-	*stx1*	423 bp	[18]
EPEC-300	R-GGAATCAGACGCAGACTGGTAGT- F-GGAAGTCAAATTCATGGGGGTAT-	*bfpA*	300 bp	[18]
ETEC-187	F-GCTAAACCAGTAGAGGTCTTCAAAA- R-CCCGGTACAGAGCAGGATTACAACA-	*estA* (ST)	187 bp	[18]
EIEC-508	R-CACACGGAGCTCCTCAGTC- F-CCCCCAGCCTAGCTTAGTTT-	*ipaH*	508 bp	[18]
EAEC-215	R-ACGACACCCCTGATAAACAA- F-ATTGTCCTCAGGCATTTCAC-	*aaiC*	215 bp	[18]

**Table 2:** Episodes of diarrhoea prior to HWTS implementation and water and sanitation per household in Makwane Village.

Characteristics		Frequency (*n* = 88)	Percentage (%)	*p*-Value
Episodes of diarrhoea	No	22	25	*p* = 0.000176115
	Yes	66	75	
Diarrhoeal episodes based on water source	Roof water harvesting	06	6.8	
	River/stream water	62	70.5	
	Open spring	12	13.6	
	Borehole	8	9.1	
	Municipal treated tap water	NA	NA	
Access to proper sanitation *	With access	52	59.1	*p* = 0.369174254
	No access	36	40.9	

* Proper sanitation was characterised by any type of latrine; NA: there were no municipal tap water in the village.

**Table 3:** Mean *E. coli* reduction, turbidity reduction, temperature, and pH of untreated and treated water from control and intervention households.

Water Quality Parameters	Control Households	Intervention Households		
	Untreated Water	BSZ-SICG	SIPP	*p*-Value
*E. coli*	4.3830 Log10 * CFU/100 mL	0.4770 Log10 CFU/100 mL	** NG	0.000004201
% *E. coli* reduction	-	89.1%	100%	
Turbidity (*** NTU)	168	2.34	0.85	0.000025949
% Turbidity reduction	-	98.6%	99.5%	
Temperature (°C)	25.0	19.8	22.5	
pH	8.2	7.8	7.5	

* CFU—colony-forming unit per 100 mL of the water sample; ** NG—no growth noted from water filtered by SIPP; *** NTU—nephelometric turbidity unit.

**Table 4:** Episodes of diarrhoea per age group and stool consistency subsequent to HWTS implementation in Makwane Village.

Characteristics		Control Households (*n* = 108)		Intervention Households (*n* = 372)		*p*-Value
Age Groups	Episodes of Diarrhoea	Frequency	Percent (%)	Frequency	Percent (%)	
0–5	Yes	17	85	4	5.1	0.0000105
	No	3	15	75	94.9	
6–12	Yes	9	60	3	4.6	0.0000959
	No	6	40	62	95.4	
13–21	Yes	7	36.8	1	1.4	0.0001004
	No	12	63.2	70	98.6	
22–55	Yes	20	47.6	3	2.3	0.0000096
	No	22	52.4	127	97.7	
≥56	Yes	9	75	3	11.1	0.0005018
	No	3	25	24	88.9	
Stool consistency	Watery	43/62	69.3	14/14	100.0	
	Bloody	6/62	9.7	0/14	0	
	Mucus	13/62	21	0/14	0	
Overall episodes of diarrhoea in control households			Overall episodes of diarrhoea in intervention households			
62/108 (57.4%)			14/372 (3.8%)			

## Supplementary Materials

The following are available online at http://www.mdpi.com/1660-4601/15/3/410/s1, Supplementary File 1: Informed consent form.
