# Supplementary material for: Dramatic Reduction in Diarrhoeal Diseases through Implementation of Cost-Effective Household Drinking Water Treatment Systems in Makwane Village, Limpopo Province, South Africa
Source: Int J Environ Res Public Health. 2018 Feb 27;15(3):410. doi: 10.3390/ijerph15030410 (PMC5876955; doi:10.3390/ijerph15030410)
Supplement: Supplementary file 1 [file ijerph-15-00410-s001.pdf]

**Supplementary material (S1)**

**Informed consent form**

(Form for research subject's permission, must be signed by each research subject, and must be kept on record by the researcher)

Title of the research project: **Implementation of cost-effective decentralized household water treatment systems for the production of adequate clean and safe drinking water in rural communities**

I ……………………………. hereby voluntarily grant my permission for participating in the project as explained to me by…………………………….

The nature, objective, possible safety and health implications have been explained to me and I understand them.

I understand my right to choose whether to participate in the project and that the information furnished will be handled confidentially. I am aware that the results of the investigation may be used for the purpose of publication.

Upon signature of this form, you will be provided with a copy.

Signature…………………………………………………… Date…………………………………….

Witness………………………………………………………… Date…………………………………

Researcher…………………………………………………… Date………………………………………
